# Masticatory Muscle Function in Growing Patients with Unilateral Posterior Crossbite: A Case–Control Study Combining Surface Electromyography and Myotonometry

**DOI:** 10.3390/dj14070426

**Published:** 2026-07-10

**Authors:** Lara Díaz-González, Carlos López-de-Celis, Albert Pérez-Bellmunt, Francisco Guinot

**Affiliations:** 1 Department of Pediatric Dentistry, Faculty of Dentistry, Universitat Internacional de Catalunya, 08195 Sant Cugat del Vallès, PC, Spain; lara.diaz.ortodoncia@gmail.com (L.D.-G.); fguinot@uic.es (F.G.); 2 Department of Physiotherapy, Faculty of Medicine and Health Sciences, Universitat Internacional de Catalunya, 08195 Sant Cugat del Vallès, PC, Spain; carlesldc@uic.es; 3 Actium Functional Anatomy Research Group, 08193 Bellaterra, PC, Spain; 4 Unit of Human Anatomy and Embryology, Department of Morphological Sciences, Faculty of Medicine, Universitat Autònoma de Barcelona, 08193 Bellaterra, PC, Spain; 5 Pediatric Dentistry Service, Hospital HM Nens, HM Hospitales, 08009 Barcelona, PC, Spain; 6 HM Hospitales Health Research Institute, 28015 Madrid, PC, Spain

**Keywords:** posterior crossbite, unilateral posterior crossbite, masticatory muscles, surface electromyography, myotonometry, muscle asymmetry, orthodontics

## Abstract

**Background**: Unilateral posterior crossbite (UPXB) is a common malocclusion in growing patients, often associated with functional mandibular deviation and asymmetric masticatory function. However, its relationship with muscle activity remains unclear. This study aimed to evaluate masticatory muscle activity and viscoelastic properties in growing patients with UPXB compared to subjects with normal occlusion. **Methods**: This case–control study included 140 growing patients (60 controls and 80 with UPXB: 36 right-side and 44 left-side). Surface electromyography (sEMG) was used to assess the activity of the superficial masseter and anterior temporalis muscles during standardized static and dynamic tasks. Muscle viscoelastic properties were evaluated using myotonometry. To minimize potential bias related to craniofacial morphology, only mesofacial subjects (Ricketts’ VERT −0.5 to +0.5) were included. Statistical significance was set at *p* < 0.05. **Results**: No differences were found in sex distribution, whereas the control group was slightly older than the UPXB group. Myotonometric analysis showed limited between-group differences, with isolated findings including higher stiffness of the non-crossbite anterior temporalis in left UPXB patients (*p* = 0.014), and minor differences in stiffness and relaxation of the left masseter between groups (*p* = 0.049; *p* = 0.045). Electromyographic results showed no significant differences during maximum voluntary contraction in intercuspation. In controls, higher activity was found in the left anterior temporalis during contraction on cotton rolls (*p* = 0.039) and in the right masseter at rest (*p* = 0.033). In left UPXB patients, the non-crossbite temporalis showed higher activity (*p* = 0.023). Increased activity of the crossbite-side left masseter was found in patients with UPXB during contraction and mastication (*p* = 0.012; *p* = 0.002). During mastication, both groups exhibited higher activity on the chewing side. **Conclusions**: UPXB is associated with specific, condition-dependent alterations in masticatory muscle activity and viscoelastic properties rather than generalized neuromuscular asymmetry. These findings suggest the presence of functional adaptations in growing patients, particularly under certain loading conditions, and highlight the importance of early diagnosis to better understand its potential impact on craniofacial development.

## 1. Introduction

Posterior crossbite (PXB) is defined as an inverted buccolingual relationship of one or more posterior teeth, in which the buccal cusp of the maxillary tooth occludes within the central fossa of the corresponding mandibular tooth [1]. It represents one of the most common malocclusions in pediatric populations, with a reported prevalence ranging from 8% to 22% among orthodontic patients in primary and mixed dentition [2,3,4,5], and from 5% to 15% in the general population [6,7,8,9,10]. PXB may be classified as bilateral or unilateral. In growing patients, unilateral posterior crossbite (UPXB) accounts for approximately 71–84% of cases and is frequently associated with functional mandibular deviation [11].

Altered occlusal relationships have been associated with differences in the condyle–fossa relationship between the right and left sides of the temporomandibular joint [12]. In patients with UPXB, asymmetric masticatory function has been linked to differences in mandibular growth between both sides [13,14,15,16], asymmetric contraction of the masticatory muscles [17,18,19,20,21], reduced masseter muscle thickness on the crossbite side [22], and altered chewing patterns [23,24,25]. These findings suggest that UPXB may contribute to the development of skeletal asymmetries and temporomandibular disorders [10,26,27].

Since maxillary transverse deficiency is considered the most common etiological factor of UPXB, early treatment with rapid maxillary expansion (RME) has been widely recommended [28,29,30]. This intervention aims to reduce the risk of developing temporomandibular disorders and craniofacial abnormalities in adulthood, which may arise from altered posture and asymmetric growth and function of skeletal and muscular structures [31,32].

Masticatory muscle activity is commonly assessed using surface electromyography (sEMG), a non-invasive technique that records the electrical activity generated during muscle contraction [33]. This method allows quantification of the summation of motor unit action potentials detected through electrodes placed on the skin surface [34]. In addition, muscle viscoelastic properties—such as tone, stiffness, and relaxation—can be evaluated using myotonometry, providing complementary information on neuromuscular function [35,36]. While sEMG primarily reflects the electrophysiological behavior of the muscles during contraction, myotonometry assesses their biomechanical and viscoelastic characteristics at rest. Therefore, the combined use of both techniques may provide a more comprehensive assessment of masticatory muscle function than either method alone. However, despite the potential complementarity of these approaches, studies simultaneously evaluating electrophysiological activity and viscoelastic masticatory muscle properties in pediatric patients remain scarce.

Previous studies have reported that masticatory muscle activity may vary depending on head posture, with a more anterior head position associated with increased activity of the masseter and anterior temporalis muscles [37,38,39,40,41]. Therefore, standardization of head position is essential to ensure reliable and comparable results.

It has also been reported that craniofacial morphology influences masticatory muscle function [42,43]. Hyperdivergent subjects tend to exhibit reduced muscle strength [43,44], decreased bite force [43,45,46], and lower electromyographic activity [47,48,49]. Consequently, controlling for facial pattern is crucial in studies evaluating muscle activity.

Despite the available evidence, the literature shows conflicting results regarding the impact of posterior crossbite and its treatment on masticatory muscle activity. Some studies have reported no significant differences in muscle symmetry between subjects with and without crossbite [50], whereas others suggest that treatment may lead to a more symmetrical activation pattern [51,52]. In contrast, other authors have found that masticatory muscle asymmetry in patients with crossbite is comparable to that of individuals without malocclusion and that correction does not significantly improve symmetry [53]. Additionally, some studies have reported normalization of muscle activation patterns following rapid maxillary expansion [54]. However, these findings should be interpreted with caution due to methodological limitations, such as the absence of untreated control groups in some studies [55,56].

Given the heterogeneity and inconsistency of previous findings, further research is needed to better understand the relationship between unilateral posterior crossbite and masticatory muscle function in growing patients. Therefore, the aim of this study was to evaluate masticatory muscle activity and viscoelastic properties in growing patients with unilateral posterior crossbite and functional mandibular deviation, compared with subjects presenting normal occlusion, using surface electromyography and myotonometry under controlled craniofacial conditions.

## 2. Materials and Methods

### 2.1. Study Design and Ethical Approval

This observational case–control study was approved by the Research Ethics Committee (CEIm) of HM Hospitales (approval No. 243), the Executive Research Commission (CEIDI) of HM Hospitales, and the Ethics Committee of the Universitat Internacional de Catalunya. The study was conducted in accordance with the Declaration of Helsinki and was registered at ClinicalTrials.gov (NCT04962685).

Written informed consent was obtained from the parents or legal guardians of all participants prior to inclusion.

### 2.2. Participants and Sample Size Calculation

The sample consisted of pediatric patients attending their first orthodontic consultation at the Dental Area of HM Nens Hospital, HM Hospitales (Barcelona, Spain) between May 2022 and May 2023.

Sample size was calculated using GRANMO® software (version 7.12; Institut Municipal d'Investigació Mèdica, Barcelona, Spain), based on the symmetry index of mastication (SMI) reported in a previous study [53]. The SMI quantifies the symmetry of masticatory muscle activity between left- and right-side chewing tasks based on surface electromyographic recordings.

Assuming a standard deviation of 20.2, a minimum detectable difference of 12.3, α = 0.05, β = 0.2 (power = 80%), and a 15% dropout rate, a minimum of 100 subjects (50 per group) was required.

### 2.3. Eligibility Criteria

#### 2.3.1. Inclusion Criteria

• Mixed or early permanent dentition;
• Complete eruption of the four first permanent molars;
• Angle Class I (±1 mm);
• Mesofacial pattern (Ricketts’ VERT = 0 ± 0.5).

#### 2.3.2. Exclusion Criteria

• Systemic diseases or craniofacial anomalies affecting growth;
• Signs or symptoms of temporomandibular disorders;
• Skeletal mandibular deviation;
• Previous head trauma that might have impaired unilateral mandibular growth;
• Dental pain;
• Previous orthodontic treatment;
• Bilateral posterior crossbite.

After enrollment according to these general eligibility criteria, participants were allocated into the following study groups based on their transverse occlusal characteristics:

• Group A (control): subjects with normal occlusion (no transverse alterations).
• Group B: subjects with unilateral posterior crossbite (UPXB) and functional mandibular deviation.
  ▪ B1: right-side UPXB;
  ▪ B2: left-side UPXB.

### 2.4. Clinical and Cephalometric Assessment

Standardized extraoral (frontal and profile, at rest and smiling) and intraoral (frontal, right and left lateral in maximum intercuspation, open mouth, and occlusal views) photographs were obtained for all participants.

In Group A, normal occlusion and Angle Class I (±1 mm) were clinically confirmed. In Group B, unilateral posterior crossbite was diagnosed by the presence of at least one posterior tooth in complete crossbite, defined as the maxillary buccal cusp occluding lingually to the mandibular buccal cusp.

Functional mandibular deviation was assessed using Dawson’s manipulation technique [57] to identify discrepancies between centric relation (CR) and maximum intercuspation (MI). Only subjects presenting a functional shift (complete crossbite in MI but incomplete in CR) were included.

A lateral cephalometric radiograph was obtained for each subject. Cephalometric analysis was performed using OrtoMed® software (version 04.7 Infomed S.L., Barcelona, Spain) according to Ricketts’ analysis.

The facial biotype was determined using the VERT index, which evaluates craniofacial morphology by relating the measurements of facial axis (FA), facial depth (FD), mandibular plane angle (MP), lower facial height (LFH), and mandibular arch (MA), according to the following formula:

VERT = [((FA patient − FA norm)/3) + ((FD patient − FD norm)/3) + ((MP norm − MP patient)/4) + ((LFH norm − LFH patient)/4) + ((MA patient − MA norm)/5)]/5

Based on the VERT value, subjects were classified into the following facial types: severe dolichofacial (−2), dolichofacial (−1), mild dolichofacial (−0.5), mesofacial (0), brachyfacial (+0.5), and severe brachyfacial (+1).

To minimize potential bias related to craniofacial morphology, only subjects with a mesofacial pattern (VERT between −0.5 and +0.5) were included in the present study.

### 2.5. Surface Electromyography (sEMG) Assessment

Bilateral activity of the superficial masseter and anterior temporalis muscles was recorded simultaneously using a wireless surface EMG system (mDurance^®^; mDurance Solutions S.L., Granada, Spain), which has demonstrated concurrent validity against a reference EMG platform in controlled testing [58]. Signals were sampled at 1024 Hz and analyzed using the manufacturer’s software, with amplitude quantified using a moving RMS window (250 ms) with 50% overlap.

After cleansing the skin with alcohol to reduce impedance, disposable bipolar surface electrodes (10 mm diameter; 20 ± 1 mm interelectrode distance) were positioned 3 cm above and anterior to the mandibular angle, with the upper pole placed at the intersection of the tragus–labial commissure and exocanthion–gonion lines to record the masseter muscle, and vertically along the anterior margin of the muscle (corresponding to the frontoparietal suture) for the anterior temporalis; a reference electrode was placed on an electrically neutral site (forehead), following the protocol described by a previous study [59]. Recordings were obtained in a standardized seated posture with the Frankfurt plane parallel to the floor, to minimize any interference of head position with muscle activity.

The task battery included resting activity (10 s, with no occlusal contact or swallowing), maximum voluntary contraction (MVC) in the intercuspal position (5 s), MVC on cotton rolls placed bilaterally from the mandibular first molar to the canine (5 s) to standardize occlusal support, and unilateral chewing trials using orthodontic wax (Vitis^®^, Dentaid SL, Barcelona, Spain) for 15 s on each side to characterize functional muscle recruitment. A minimum rest period of 2 min was allowed between tasks to minimize muscle fatigue.

### 2.6. Myotonometric Assessment

Muscle viscoelastic properties were quantified bilaterally in the superficial masseter and anterior temporalis muscles using a handheld myotonometer (MyotonPro^®^; Myoton Ltd., Tallinn, Estonia). The device delivers a brief, low-force mechanical impulse (15 ms; ~0.4 N) under a controlled pre-compression load (~0.18 N), generating damped oscillations from which objective parameters are derived.

Participants were examined in a standardized seated posture with the head in a natural, neutral position and the mandible in a relaxed rest posture (lips lightly together, teeth not in contact) to approximate passive muscle conditions. The point of maximal muscle belly prominence was identified by palpation during a brief submaximal clench, marked on the skin, and the probe was applied perpendicular (±5°) to the marked point.

Three consecutive measurements at rest were recorded at 1 s intervals, and mean values were calculated for oscillation frequency (F, Hz), dynamic stiffness (S, N/m), decrement (D), and mechanical stress relaxation time (R, ms), representing muscle tone, mechanical resistance to deformation, elasticity-related properties, and viscoelastic recovery capacity, respectively.

This approach is supported by published protocols that report reference values and minimal detectable change thresholds for MyotonPro-derived parameters, and by reliability studies showing good-to-excellent reproducibility for cervico-mandibular and masticatory muscles [36,60].

### 2.7. Calibration and Reliability Assessment

All procedures were performed by two operators. Operator 1 was responsible for clinical examination, photographic records, and cephalometric analysis, while Operator 2 performed surface electromyography (sEMG) and myotonometric measurements.

Prior to data collection, both operators underwent a calibration process to ensure methodological consistency. For Operator 1, thirty orthodontic patients were randomly selected. These subjects were not part of the study sample and were recruited exclusively for calibration purposes. Intraoral photographs were independently evaluated by Operator 1 and an experienced orthodontist, classifying subjects into three diagnostic categories (no crossbite, unilateral crossbite, or bilateral crossbite). Inter-examiner agreement was high (Cohen’s kappa = 0.81–1.00).

For Operator 2, intra-operator reliability was assessed by repeating the full sEMG and myotonometric protocol in five subjects at two time points separated by 1–4 weeks.

In addition, equipment calibration and standardization procedures were applied prior to each measurement session according to the manufacturer’s recommendations. For sEMG, electrode placement and signal acquisition parameters were standardized across sessions, and impedance was verified before each recording. For myotonometry, the device was calibrated before use following the manufacturer’s built-in calibration protocol.

### 2.8. Statistical Analysis

Statistical analysis was performed using SPSS v.20 statistical package (IBM Corp., Armonk, NY, USA). Data normality was assessed using the Kolmogorov–Smirnov test. Although some variables showed deviations from a normal distribution, parametric tests were used due to the documented robustness of the ANOVA model against non-normality [61,62], particularly in balanced designs with a sufficiently large sample size (n = 140). Descriptive statistics were performed for all variables, with quantitative data expressed as mean ± standard deviation (SD) and categorical data as absolute frequencies and percentages.

To compare categorical variables, such as sex, Fisher’s exact test was employed due to its precision in small subgroup distributions. Differences in age were analyzed using a one-way analysis of variance (ANOVA). The core analysis of muscular function was performed using a two-way mixed ANOVA (3 × 2) to evaluate the interaction between Group (inter-subject factor: control, right crossbite, or left crossbite) and Side (intra-subject factor: right, left) on myotonometry and electromyography outcomes. This model was chosen specifically to detect whether the effect of the malocclusion (UPXB) differed between sides within each group, an interaction that non-parametric alternatives cannot adequately assess.

Effect sizes were estimated using Cohen’s d coefficient and interpreted as small (d < 0.1), medium (d < 0.3), or large (d < 0.7) [63]. The statistical significance level was set at *p* < 0.05 for all tests.

## 3. Results

To increase statistical power and account for subgroup analyses (right and left UPXB), a larger sample than the minimum required was recruited. The final sample consisted of 60 patients in Group A (normal occlusion) and 80 patients in Group B with unilateral posterior crossbite (36 right-side and 44 left-side). The distribution of the sample according to occlusion group, sex, and age is presented in Table 1. No statistically significant differences were observed in sex distribution (*p* = 0.319). However, Group A showed a higher mean age than both the right UPXB group (mean difference: 1.1 years; *p* = 0.015) and the left UPXB group (mean difference: 1.7 years; *p* = 0.001), while no differences were found between right and left UPXB groups (*p* = 0.341).

**Figure 1:**
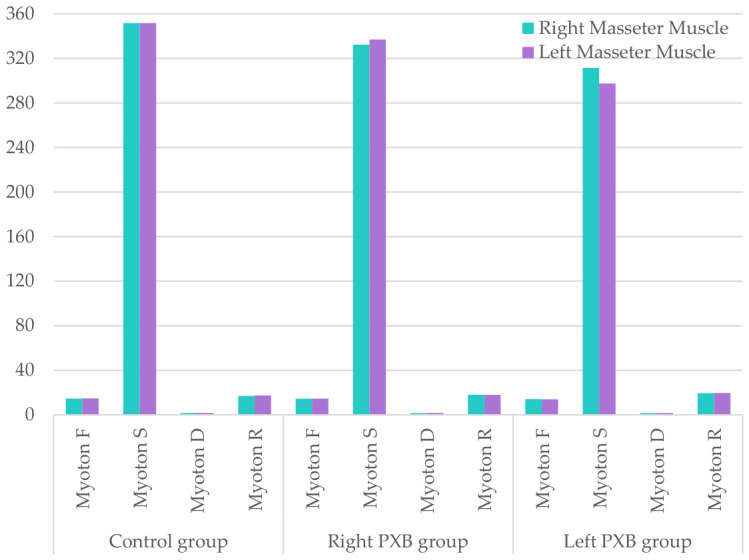
Myotonometric measurements of the masseter muscle (right vs. left).

**Figure 2:**
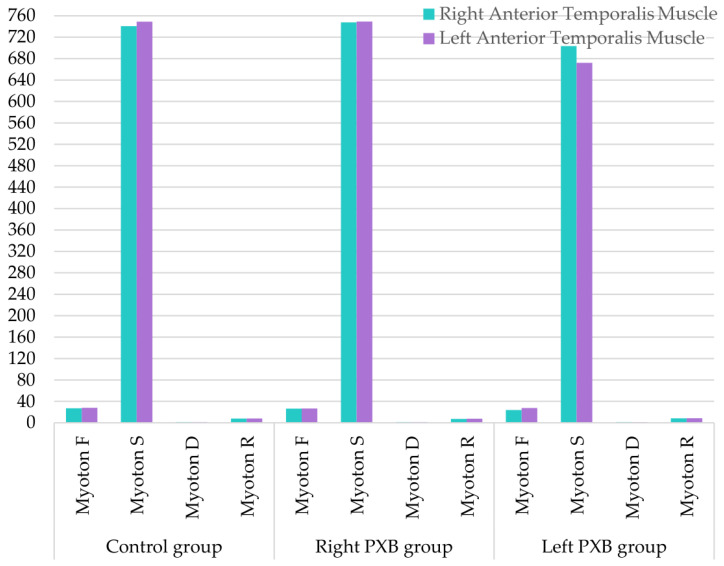
Myotonometric measurements of the anterior temporalis muscle (right vs. left).

Myotonometric measurements of the masseter and anterior temporalis muscles are presented in Figure 1 and Figure 2, respectively, while the complete numerical data comparing right and left sides within each group are provided in the Supplementary Material (Tables S1 and S2). Overall, few statistically significant differences were observed. Notably, in patients with left UPXB, the non-crossbite anterior temporalis muscle (right side) exhibited significantly higher stiffness compared to the crossbite side (*p* = 0.014), whereas no other significant side differences were detected.

Between-group comparisons of myotonometric parameters are presented in Table 2. The stiffness of the left masseter muscle was significantly higher in the control group than in the left UPXB group (*p* = 0.049). In contrast, relaxation values of the crossbite-side left masseter were significantly higher in the UPXB group compared to controls (*p* = 0.045), while no additional significant differences were identified.

**Figure 3:**
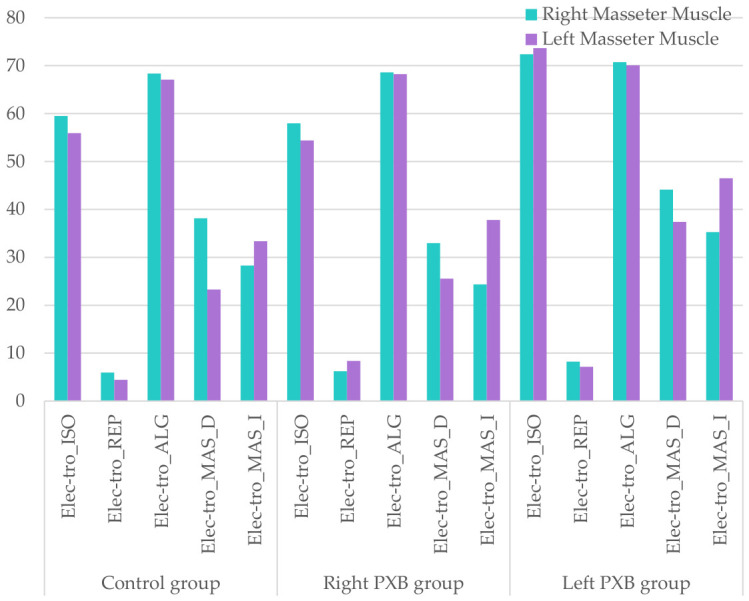
Comparison of electromyographic activity (RMS mean values) of the masseter muscle (right vs. left).

**Figure 4:**
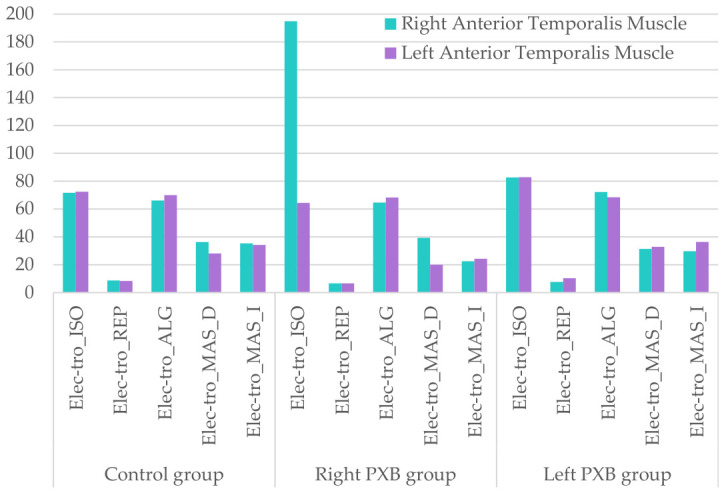
Comparison of electromyographic activity (RMS mean values) of the anterior temporalis muscle (right vs. left).

Surface electromyography results are presented in Figure 3 and Figure 4 for the masseter and anterior temporalis muscles, respectively, while the complete numerical data are provided in the Supplementary Material (Tables S3 and S4). In the control group, no significant side differences were observed during maximum voluntary contraction in intercuspation. However, during contraction on cotton rolls, the left anterior temporalis showed significantly higher activity than the right (*p* = 0.039), whereas at rest, the right masseter exhibited higher activity than the left (*p* = 0.033). In the left UPXB group, the non-crossbite anterior temporalis (right side) demonstrated significantly higher activity during cotton roll clenching (*p* = 0.023), while no differences were observed at rest or during maximum voluntary contraction in intercuspation. During mastication, both groups showed increased activity of the masseter and anterior temporalis muscles on the chewing side.

Between-group comparisons of electromyographic activity are presented in Table 3. The right masseter muscle, regardless of whether it was on the crossbite or non-crossbite side, showed no significant differences compared to the control group, and similar findings were observed for the non-crossbite left masseter. In contrast, the crossbite-side left masseter showed significantly higher activity in UPXB patients than in controls during maximum voluntary contraction (difference: −17.52 ± 6.07; *p* = 0.012) and during right-side mastication (difference: −14.12 ± 4.10; *p* = 0.002). No significant differences were observed for the anterior temporalis muscle between the control and UPXB groups.

When comparing the right and left UPXB groups, differences were identified primarily in the masseter muscle. During left-side mastication, the crossbite-side right masseter showed lower activity than the non-crossbite side (difference: −10.89 ± 4.28; *p* = 0.036), whereas during right-side mastication, the non-crossbite left masseter exhibited lower activity than the crossbite side (difference: −11.82 ± 4.64; *p* = 0.036). Additionally, during maximum voluntary contraction, the non-crossbite left masseter showed lower activity compared to the crossbite side (difference: −19.26 ± 6.87; *p* = 0.017). Regarding the anterior temporalis muscle, a significant difference was observed during left-side mastication, with lower activity on the non-crossbite side than on the crossbite side (difference: −12.77 ± 5.04; *p* = 0.037).

## 4. Discussion

The present study aimed to evaluate masticatory muscle activity and viscoelastic properties in growing patients with unilateral posterior crossbite (UPXB) and functional mandibular deviation, compared to subjects with normal occlusion. The results indicate that UPXB is associated with specific and condition-dependent neuromuscular alterations rather than a generalized or consistent muscle asymmetry.

Regarding the myotonometric findings, only limited differences were observed between groups and sides. The increased stiffness of the non-crossbite anterior temporalis muscle in patients with left UPXB, as well as the differences in stiffness and relaxation of the left masseter between control and crossbite groups, suggest localized adaptations of muscle mechanical properties. These findings are partially consistent with previous studies reporting structural and functional alterations in masticatory muscles in patients with posterior crossbite, such as reduced masseter thickness on the affected side [22] and altered muscle contraction patterns [17,18,19,20,21]. However, the absence of consistent bilateral differences in the present study suggests that these changes may be subtle and influenced by functional demands rather than representing a stable neuromuscular imbalance.

The electromyographic results further support the hypothesis of task-dependent asymmetry. No side differences were observed during maximum voluntary contraction in intercuspation, in agreement with previous studies reporting similar muscle activation patterns in individuals with and without crossbite [50,53]. However, asymmetries emerged under specific conditions, such as rest and cotton-roll clenching. In the control group, higher activity was observed in the left anterior temporalis during cotton roll clenching and in the right masseter at rest, which is in line with previous evidence showing that mild physiological asymmetries can be present even in individuals with normal occlusion [59,64].

In patients with left UPXB, the non-crossbite anterior temporalis showed higher activity during cotton roll clenching, suggesting a compensatory functional mechanism favoring the non-affected side. This observation is consistent with previous reports indicating that neuromuscular adaptation may occur in response to occlusal discrepancies [19,20,65]. Furthermore, studies evaluating the effects of treatment have suggested that correction of posterior crossbite may lead to a more symmetrical activation pattern [51,52], although this remains controversial. Other authors have reported minimal or no differences in muscle symmetry between crossbite and control groups [50,53], reinforcing the variability observed in the literature.

During mastication, both control and UPXB groups showed higher muscle activity on the chewing side, reflecting a normal physiological response. However, between-group comparisons revealed increased activation of the crossbite-side left masseter in UPXB patients during both maximum voluntary contraction and mastication. This finding suggests that the crossbite side may be subjected to greater functional loading under specific conditions. Previous studies have associated unilateral crossbite with asymmetric mandibular growth and temporomandibular joint adaptation [13,14,15,16], as well as altered chewing patterns [23,24,25], supporting the hypothesis that functional asymmetries may influence craniofacial development over time.

The absence of consistent asymmetry across all tasks challenges the traditional assumption that UPXB is invariably associated with a generalized neuromuscular imbalance. Instead, the present findings support the hypothesis that neuromuscular adaptation may be dynamic and task-dependent, potentially reflecting compensatory mechanisms rather than pathological dysfunction. This may explain the discrepancies in the literature regarding the impact of crossbite and its treatment on masticatory muscle activity [50,51,52,53,54]. Methodological differences, including variability in study design, sample characteristics, and absence of control groups in some studies [55,56], may further contribute to these inconsistent findings.

An important strength of the present study is the combined use of surface electromyography and myotonometry, allowing a comprehensive assessment of both muscle activity and mechanical properties. Additionally, the inclusion of only mesofacial subjects minimized the influence of craniofacial morphology, which has been shown to significantly affect muscle function, bite force, and electromyographic activity [42,43,44,45,46,47,48,49,66]. Standardization of head posture during recordings further enhanced the reliability and comparability of the results, as head position has been demonstrated to influence masticatory muscle activity [37,38,39,40,41]. The large sample size and the consistency of the testing protocol further strengthen the reliability of the findings.

From a clinical perspective, the present findings suggest that UPXB in growing patients is associated with subtle and condition-dependent functional adaptations rather than pronounced or generalized muscle asymmetry. Importantly, the absence of consistent differences during intercuspal maximum voluntary contraction suggests that conventional static assessments may not be sufficiently sensitive to detect subtle functional alterations, which appear more evident under specific task-dependent conditions. Although early treatment of posterior crossbite has been recommended to prevent long-term skeletal and functional alterations [28,29,30], the present results indicate that neuromuscular differences alone should not be considered a determinant for treatment timing. Instead, clinical decision-making should integrate skeletal, occlusal, and developmental factors, as well as functional assessments under different loading conditions. Further longitudinal and interventional studies are required to determine whether these functional adaptations persist over time or are modified following orthodontic treatment.

Despite the strengths of the present study, several limitations should be acknowledged. The cross-sectional design precludes the establishment of causal relationships between unilateral posterior crossbite and neuromuscular alterations. Although the sample size was adequate, the age differences observed between groups may have influenced muscle activity and viscoelastic properties. In addition, only the superficial masseter and anterior temporalis muscles were evaluated, which may not fully represent the complexity of the masticatory system. The absence of longitudinal follow-up also limits the ability to determine whether the observed adaptations persist over time or change after orthodontic treatment. Furthermore, functional assessments were performed under controlled clinical conditions, which may not entirely reflect natural chewing behavior. Future studies should include longitudinal designs, larger and more homogeneous samples, and a broader assessment of masticatory function to better understand the clinical implications of these findings.

## 5. Conclusions

• Growing patients with unilateral posterior crossbite and functional mandibular deviation exhibited specific, condition-dependent alterations in masticatory muscle activity and viscoelastic properties, rather than a consistent or generalized neuromuscular asymmetry.
• Myotonometric findings revealed limited differences between groups, suggesting that muscle mechanical properties are only moderately affected and may reflect localized adaptations. Electromyographic results demonstrated that muscle asymmetries were not evident during maximum voluntary contraction in intercuspation but emerged under specific functional conditions, such as rest, cotton roll clenching, and mastication. In particular, increased activation of the crossbite-side masseter under loading conditions suggests a functional adaptation to occlusal imbalance.
• These findings indicate that unilateral posterior crossbite in growing patients is associated with subtle neuromuscular adaptations rather than marked dysfunction. From a clinical perspective, this supports the concept that functional alterations may precede or contribute to structural changes during growth, although their magnitude appears limited.
• Further longitudinal and interventional studies are needed to determine whether these neuromuscular adaptations persist over time and to clarify their clinical relevance in relation to craniofacial development and the timing of orthodontic treatment.

## Figures and Tables

**Table 1:** Sample distribution according to group, sex, and age.

	A: Normal Occlusion (Control Group) (n = 60)	B1: Right Posterior Crossbite (n = 36)	B2: Left Posterior Crossbite (n = 44)
Sex			
Male	27 (45%)	20 (55.6%)	17 (38.6%)
Female	33 (55%)	16 (44.4%)	27 (61.4%)
Age (years)	10.1 ± 2.1	9.0 ± 1.4	8.4 ± 1.8

**Table 2:** Between-group comparison of myotonometric parameters of the masseter and anterior temporalis muscles in control and unilateral posterior crossbite groups.

	Difference Control Group—Right PXB Group		Difference Control Group—Left PXB Group		Difference Right PXB Group—Left PXB Group	
	Media ± SD	*p*	Media ± SD	*p*	Media ± SD	*p*
RMM						
Myoton F	0.07 ± 0.57	1.000	0.50 ± 0.53	1.000	0.42 ± 0.61	1.000
Myoton S	19.32 ± 26.24	1.000	40.26 ± 24.70	0.316	20.95 ± 27.97	1.000
Myoton D	0.02 ± 0.06	1.000	0.04 ± 0.06	1.000	0.02 ± 0.06	1.000
Myoton R	−1.09 ± 1.10	0.980	−2.36 ± 1.04	0.074	−1.27 ± 1.18	0.842
LMM						
Myoton F	0.26 ± 0.50	1.000	0.98 ± 0.47	0.115	0.72 ± 0.53	0.526
Myoton S	14.83 ± 23.71	1.000	54.27 ± 22.32	0.049	39.44 ± 25.27	0.363
Myoton D	0.02 ± 0.06	1.000	0.07 ± 0.05	0.622	0.05 ± 0.06	1.000
Myoton R	−0.64 ± 1.05	1.000	−2.43 ± 0.99	0.045	−1.79 ± 1.12	0.333
RATM						
Myoton F	0.71 ± 1.21	1.000	3.33 ± 1.14	0.012	2.62 ± 1.29	0.132
Myoton S	−6.98 ± 25.11	1.000	37.31 ± 23.64	0.350	44.29 ± 26.77	0.301
Myoton D	0.00 ± 0.05	1.000	−0.02 ± 0.04	1.000	−0.02 ± 0.05	1.000
Myoton R	0.66 ± 0.48	0.503	−0.31 ± 0.45	1.000	−0.98 ± 0.51	0.173
LATM						
Myoton F	1.22 ± 3.26	1.000	0.25 ± 3.07	1.000	−0.96 ± 3.48	1.000
Myoton S	−0.34 ± 27.07	1.000	76.65 ± 25.48	1.000	76.99 ± 28.85	0.026
Myoton D	0.05 ± 0.04	0.794	0.07 ± 0.04	0.276	0.02 ± 0.05	1.000
Myoton R	0.30 ± 0.41	1.000	−0.43 ± 0.39	0.814	−0.73 ± 0.44	0.297

Abbreviations: RMM: Right Masseter Muscle; LMM: Left Masseter Muscle; RATM: Right Anterior Temporalis Muscle; LATM: Left Anterior Temporalis Muscle; PXB: Posterior Crossbite; SD: Standard Deviation; F: Frequency (Hz); S: Stiffness (Nw/m); D: Decrement R: Relaxation (ms).

**Table 3:** Between-group comparison of electromyographic activity (RMS values) of the masseter and anterior temporalis muscles among control, right posterior crossbite, and left posterior crossbite groups.

	Difference Control—Right PXB		Difference Control—Left PXB		Difference Right PXB—Left PXB	
	Mean ± SD	*p*	Mean ± SD	*p*	Mean ± SD	*p*
RMM						
Electro_ISO_RMS_mean	1.51 ± 7.12	1.000	−12.89 ± 6.71	0.170	−14.41 ± 7.59	0.180
Electro_REP_RMS_mean	−0.26 ± 1.61	1.000	−2.27 ± 1.51	0.411	−2.00 ± 1.72	0.735
Electro_ALG_RMS_mean	−0.25 ± 2.46	1.000	−2.68 ± 2.31	0.914	−2.14 ± 2.62	1.000
Electro_MAS_D_RMS_mean	5.19 ± 4.49	0.751	−5.96 ± 4.23	0.483	−11.15 ± 4.79	0.064
Electro_MAS_I_RMS_mean	3.92 ± 4.01	0.993	−6.97 ± 3.78	0.202	−10.89 ± 4.28	0.036
LMM						
Electro_ISO_RMS_mean	1.54 ± 6.44	1.000	−17.52 ± 6.07	0.012	−19.26 ± 6.87	0.017
Electro_REP_RMS_mean	−3.93 ± 1.97	0.144	−2.72 ± 1.86	0.435	1.21 ± 2.10	1.000
Electro_ALG_RMS_mean	−1.16 ± 2.63	1.000	−3.03 ± 2.47	0.668	−1.87 ± 2.80	1.000
Electro_MAS_D_RMS_mean	−2.30 ± 4.36	1.000	−14.12 ± 4.10	0.002	−11.82 ± 4.64	0.036
Electro_MAS_I_RMS_mean	−4.44 ± 6.11	1.000	−13.15 ± 5.75	0.072	−8.71 ± 6.51	0.550
RATM						
Electro_ISO_RMS_mean	−123.21 ± 82.10	0.407	−11.01 ± 77.30	1.000	112.20 ± 87.52	0.606
Electro_REP_RMS_mean	1.97 ± 2.14	1.000	0.98 ± 2.02	1.000	−0.99 ± 2.28	1.000
Electro_ALG_RMS_mean	1.44 ± 2.80	1.000	−6.13 ± 2.63	0.064	−7.57 ± 2.98	0.037
Electro_MAS_D_RMS_mean	−3.05 ± 10.79	1.000	4.94 ± 10.16	1.000	7.99 ± 11.51	1.000
Electro_MAS_I_RMS_mean	12.88 ± 7.07	0.212	5.56 ± 6.65	1.000	−7.32 ± 7.53	1.000
LATM						
Electro_ISO_RMS_mean	7.99 ± 8.86	1.000	−10.38 ± 8.34	0.645	−18.37 ± 9.44	0.161
Electro_REP_RMS_mean	1.65 ± 2.66	1.000	−2.02 ± 2.50	1.000	−3.68 ± 2.83	0.590
Electro_ALG_RMS_mean	1.70 ± 2.58	1.000	1.52 ± 2.43	1.000	−0.18 ± 2.75	1.000
Electro_MAS_D_RMS_mean	8.01 ± 4.72	0.277	−4.76 ± 4.45	0.859	−12.77 ± 5.04	0.037
Electro_MAS_I_RMS_mean	9.91 ± 4.67	0.107	−2.14 ± 4.40	1.000	−12.04 ± 4.98	0.051

Abbreviations: RMM: Right Masseter Muscle; LMM: Left Masseter Muscle; RATM: Right Anterior Temporalis Muscle; LATM: Left Anterior Temporalis Muscle; PXB: Posterior Crossbite; SD: Standard Deviation; ISO: maximum voluntary contraction; REP: muscular activity at rest; ALG: maximum voluntary contraction on cotton rolls; MAS_D: right side mastication; MAS_I: left side mastication.

## Data Availability

The data presented in this study are available on request from the corresponding author.

## Supplementary Materials

The following supporting information can be downloaded at: https://www.mdpi.com/article/10.3390/dj14070426/s1. Table S1: Myotonometric measurements of the masseter muscle (right vs. left). Table S2: Myotonometric measurements of the anterior temporalis muscle (right vs. left). Table S3: Comparison of electromyographic activity (RMS values) of the masseter muscle between right and left sides within each study group. Table S4: Comparison of electromyographic activity (RMS values) of the anterior temporalis muscle between right and left sides within each study group.

## References

[1] Daskalogiannakis J. (2000). Glossary of Orthodontic Terms.

[2] Shalish M., Gal A., Brin I., Zini A., Ben-Bassat Y. (2013). Prevalence of dental features that indicate a need for early orthodontic treatment. Eur. J. Orthod..

[3] Sonnesen L., Bakke M., Solow B. (1998). Malocclusion traits and symptoms of temporomandibular disorders in children. Eur. J. Orthod..

[4] Melink S., Vagner M.V., Hocevar-Boltezar I., Ovsenik M. (2010). Posterior crossbite in deciduous dentition and its associations. Am. J. Orthod. Dentofac. Orthop..

[5] de Sousa R.V., Ribeiro G.L., Firmino R.T., Martins C.C., Granville-Garcia A.F., Paiva S.M. (2014). Prevalence and factors for anterior open bite and posterior crossbite. Braz. Dent. J..

[6] Thilander B., Myrberg N. (1973). The prevalence of malocclusion in Swedish schoolchildren. Scand. J. Dent. Res..

[7] Helm S., Prydsö U. (1979). Prevalence of malocclusion in medieval and modern Danes contrasted. Scand. J. Dent. Res..

[8] Ciuffolo F., Manzoli L., D’Attilio M., Tecco S., Muratore F., Festa F., Romano F. (2005). Prevalence and distribution by gender of occlusal characteristics in a sample of Italian secondary school students: A cross-sectional study. Eur. J. Orthod..

[9] Farella M., Michelotti A., Iodice G., Milani S., Martina R. (2007). Unilateral posterior crossbite is not associated with TMJ clicking in young adolescents. J. Dent. Res..

[10] Iodice G., Danzi G., Cimino R., Paduano S., Michelotti A. (2013). Association between posterior crossbite, masticatory muscle pain, and disc displacement: A systematic review. Eur. J. Orthod..

[11] Thilander B., Lennartsson B. (2002). A study of children with unilateral posterior crossbite, treated and untreated, in the deciduous dentition—occlusal and skeletal characteristics of significance in predicting the long-term outcome. J. Orofac. Orthop..

[12] Takada J., Miyamoto J.J., Yokota T., Ono T., Moriyama K. (2015). Comparison of the mandibular hinge axis in adult patients with facial asymmetry with and without posterior unilateral crossbite. Eur. J. Orthod..

[13] Hesse K.L., Artun J., Joondeph D.R., Kennedy D.B. (1997). Changes in condylar position and occlusion associated with maxillary expansion for correction of functional unilateral posterior crossbite. Am. J. Orthod. Dentofac. Orthop..

[14] Nerder P.H., Bakke M., Solow B. (1999). The functional shift of the mandible in unilateral posterior crossbite and the adaptation of the temporomandibular joints: A pilot study. Eur. J. Orthod..

[15] Pinto A.S., Buschang P.H., Throckmorton G.S., Chen P. (2001). Morphological and positional asymmetries of young children with functional unilateral posterior crossbite. Am. J. Orthod. Dentofac. Orthop..

[16] Uysal T., Sisman Y., Kurt G., Ramoglu S.I. (2009). Condylar and ramal vertical asymmetry in unilateral and bilateral posterior crossbite patients and a normal occlusion sample. Am. J. Orthod. Dentofac. Orthop..

[17] Troelstrup B., Moller E. (1970). Electromyography of the temporalis and masseter muscles in children with unilateral cross-bite. Scand. J. Dent. Res..

[18] Ingervall B., Thilander B. (1975). Activity of temporal and masseter muscles in children with a lateral forced bite. Angle Orthod..

[19] Ferrario V.F., Sforza C., Serrao G. (1999). The influence of crossbite on the coordinated electromyographic activity of human masticatory muscles during mastication. J. Oral Rehabil..

[20] Alarcón J.A., Martín C., Palma J.C. (2000). Effect of unilateral posterior crossbite on the electromyographic activity of human masticatory muscles. Am. J. Orthod. Dentofac. Orthop..

[21] Alarcón J.A., Martín C., Palma J.C., Menéndez-Núñez M. (2009). Activity of jaw muscles in unilateral cross-bite without mandibular shift. Arch. Oral Biol..

[22] Kiliaridis S., Mahboubi P.H., Raadsheer M.C., Katsaros C. (2007). Ultrasonographic thickness of the masseter muscle in growing individuals with unilateral crossbite. Angle Orthod..

[23] Nie Q., Kanno Z., Xu T., Lin J., Soma K. (2010). Clinical study of frontal chewing patterns in various crossbite malocclusions. Am. J. Orthod. Dentofac. Orthop..

[24] Sever E., Marion L., Ovsenik M. (2010). Relationship between masticatory cycle morphology and unilateral crossbite in the primary dentition. Eur. J. Orthod..

[25] Piancino M.G., Comino E., Talpone F., Vallelonga T., Frongia G., Bracco P. (2012). Reverse-sequencing chewing patterns evaluation in anterior versus posterior unilateral crossbite patients. Eur. J. Orthod..

[26] Iodice G., Danzi G., Cimino R., Paduano S., Michelotti A. (2016). Association between posterior crossbite, skeletal, and muscle asymmetry: A systematic review. Eur. J. Orthod..

[27] Michelotti A., Iodice G., Piergentili M., Farella M., Martina R. (2016). Incidence of temporomandibular joint clicking in adolescents with and without unilateral posterior cross-bite: A 10-year follow-up study. J. Oral Rehabil..

[28] Bucci R., D’Antò V., Rongo R., Valletta R., Martina R., Michelotti A. (2016). Dental and skeletal effects of palatal expansion techniques: A systematic review of the current evidence from systematic reviews and meta-analyses. J. Oral Rehabil..

[29] Rongo R., D’Antò V., Bucci R., Polito I., Martina R., Michelotti A. (2017). Skeletal and dental effects of Class III orthopaedic treatment: A systematic review and meta-analysis. J. Oral Rehabil..

[30] Agostino P., Ugolini A., Signori A., Silvestrini-Biavati A., Harrison J.E., Riley P. (2014). Orthodontic treatment for posterior crossbites. Cochrane Database Syst. Rev..

[31] Castelo P.M., Pereira L.J., Andrade A.S., Marquezin M.C., Gavião M.B. (2010). Evaluation of facial asymmetry and masticatory muscle thickness in children with normal occlusion and functional posterior crossbite. Minerva Stomatol..

[32] Veli I., Uysal T., Ozer T., Ucar F.I., Eruz M. (2011). Mandibular asymmetry in unilateral and bilateral posterior crossbite patients using cone-beam computed tomography. Angle Orthod..

[33] Pérez-Bellmunt A., Llurda L., Simon M., Navarro R., Casasayas O., López-de-Celis C., Seijas R., Álvarez P. (2019). Neuromuscular response: What is it and how to measure it?. Phys. Med. Rehabil. J..

[34] Rampichini S., Vieira T.M., Castiglioni P., Merati G. (2020). Complexity analysis of surface electromyography for assessing the myoelectric manifestation of muscle fatigue: A review. Entropy.

[35] Rodica P., Ortanescu D., Lica E., Cosma G., Rusu L. (2017). Monitoring the muscle training by evaluation of viscoelastic parameters. J. Phys. Educ. Sport.

[36] Campos López A., Estébanez-de-Miguel E., Camou Acedo T., Garcia-Pelagio K.P., Albarova-Corral I., Malo Urries M., Villanueva-Meléndez P. (2026). Reliability of myotonometry in the assessment of cervico-mandibular musculature: Inter- and intra-examiner and inter- and intra-session reliability study. CRANIO.

[37] Gadotti I.C., Berzin F., Biasotto-Gonzalez D. (2005). Preliminary rapport on head posture and muscle activity in subjects with class I and II. J. Oral Rehabil..

[38] Ohmure H., Miyawaki S., Nagata J., Ikeda K., Yamasaki K., Al-Kalaly A. (2008). Influence of forward head posture on condylar position. J. Oral Rehabil..

[39] Goldstein D.F., Kraus S.L., Williams W.B., Glasheen-Wray M. (1984). Influence of cervical posture on mandibular movement. J. Prosthet. Dent..

[40] Forsberg C.M., Hellsing E., Linder-Aronson S., Sheikholeslam A. (1985). EMG activity in neck and masticatory muscles in relation to extension and flexion of the head. Eur. J. Orthod..

[41] Gadotti I., Hicks K., Koscs E., Lynn B., Estrazulas J., Civitella F. (2020). Electromyography of the masticatory muscles during chewing in different head and neck postures—A pilot study validation. J. Oral Biol. Craniofacial Res..

[42] Throckmorton G.S., Finn R.A., Bell W.H. (1980). Biomechanics of differences in lower facial height. Am. J. Orthod..

[43] García-Morales P., Buschang P.H., Throckmorton G.S., English J.D. (2003). Maximum bite force, muscle efficiency and mechanical advantage in children with vertical growth patterns. Eur. J. Orthod..

[44] Farias S., Custodio W., Faot F., Del Bel Cury A., Rodrigues Garcia R. (2010). Masticatory features, EMG activity and muscle effort of subjects with different facial patterns. J. Oral Rehabil..

[45] Raadsheer M.C., van Eijden T.M., van Ginkel F.C., Prahl-Andersen B. (1999). Contribution of jaw muscle size and craniofacial morphology to human bite force magnitude. J. Dent. Res..

[46] Sondang P., Kumagai H., Tanaka E., Ozaki H., Nikawa H., Tanne K. (2003). Correlation between maximum bite force and craniofacial morphology of young adults in Indonesia. J. Oral Rehabil..

[47] Ueda H.M., Miyamoto K., Saifuddin M.D., Ishizuka Y., Tanne K. (2000). Masticatory muscle activity in children and adults with different facial types. Am. J. Orthod. Dentofac. Orthop..

[48] Tecco S., Caputi S., Tete S., Orsini G., Festa F. (2007). Electromyographic activity of masticatory, neck and trunk muscles of subjects with different mandibular divergence: A cross-sectional evaluation. Angle Orthod..

[49] Li H., Cui C., Lu S., He K. (2008). Study on the association of ultrasonographic thickness and electromyographic activity of masseter muscle in young females with different vertical craniofacial morphology. Shanghai J. Stomatol..

[50] Martín C., Palma J.C., Alamán J.M., López-Quiñones J.M., Alarcón J.A. (2012). Longitudinal evaluation of sEMG of masticatory muscles and kinematics of mandible changes in children treated for unilateral cross-bite. J. Electromyogr. Kinesiol..

[51] Kecik D., Kocadereli I., Saatci I. (2007). Evaluation of the treatment changes of functional posterior crossbite in the mixed dentition. Am. J. Orthod. Dentofac. Orthop..

[52] Piancino M.G., Falla D., Merlo A., Vallelonga T., de Biase C., Dalessandri D., Debernardi C. (2016). Effects of therapy on masseter activity and chewing kinematics in patients with unilateral posterior crossbite. Arch. Oral Biol..

[53] Michelotti A., Rongo R., Valentino R., D’Antò V., Bucci R., Danzi G., Cioffi I. (2019). Evaluation of masticatory muscle activity in patients with unilateral posterior crossbite before and after rapid maxillary expansion. Eur. J. Orthod..

[54] Spolaor F., Mason M., De Stefani A., Bruno G., Surace O., Guiotto A., Sawacha Z. (2020). Effects of rapid palatal expansion on chewing biomechanics in children with malocclusion: A surface electromyography study. Sensors.

[55] De Rossi M., De Rossi A., Hallak J.E., Vitti M., Regalo S.C. (2009). Electromyographic evaluation in children having rapid maxillary expansion. Am. J. Orthod. Dentofac. Orthop..

[56] Maffei C., Garcia P., de Biase N.G., de Souza Camargo E., Vianna-Lara M.S., Grégio A.M., Azevedo-Alanis L.R. (2014). Orthodontic intervention combined with myofunctional therapy increases electromyographic activity of masticatory muscles in patients with skeletal unilateral posterior crossbite. Acta Odontol. Scand..

[57] Dawson P.E. (1995). New definition for relating occlusion to varying conditions of the temporomandibular joint. J. Prosthet. Dent..

[58] Molina-Molina A., Ruiz-Malagón E.J., Carrillo-Pérez F., Roche-Seruendo L.E., Damas M., Banos O., García-Pinillos F. (2020). Validation of mDurance, a wearable surface electromyography system for muscle activity assessment. Front. Physiol..

[59] Ferrario V.F., Sforza C., Colombo A., Ciusa V. (2000). An electromyographic investigation of masticatory muscles symmetry in normo-occlusion subjects. J. Oral Rehabil..

[60] Gałczyńska-Rusin M., Pobudek-Radzikowska M., Maciejewska-Szaniec Z., Przystańska A., Czajka-Jakubowska A. (2025). Evaluating the reliability of myotonometry for assessing masseter muscle hypertrophy in healthy subjects. J. Oral Facial Pain Headache.

[61] Blanca M.J., Alarcón R., Arnau J., Bono R., Bendayan R. (2017). Non-normal data: Is ANOVA still a valid option?. Psicothema.

[62] Schmider E., Ziegler M., Danay E., Beyer L., Bühner M. (2010). Is it really robust? Reinvestigating the robustness of ANOVA against violations of the normal distribution assumption. Methodology.

[63] Zieliński G., Gawda P. (2025). Defining effect size standards in temporomandibular joint and masticatory muscle research. Med. Sci. Monit..

[64] Ferrario V.F., Tartaglia G.M., Galletta A., Grassi G.P., Sforza C. (2006). The influence of occlusion on jaw and neck muscle activity: A surface EMG study in healthy young adults. J. Oral Rehabil..

[65] Andrade A.S., Gavião M.B., Gameiro G.H., De Rossi M. (2010). Characteristics of masticatory muscles in children with unilateral posterior crossbite. Braz. Oral Res..

[66] Charalampidou M., Kjellberg H., Georgiakaki I., Kiliaridis S. (2008). Masseter muscle thickness and mechanical advantage in relation to vertical craniofacial morphology in children. Acta Odontol. Scand..

