# Supplementary material for: Masticatory Muscle Function in Growing Patients with Unilateral Posterior Crossbite: A Case–Control Study Combining Surface Electromyography and Myotonometry
Source: Dent J (Basel). 2026 Jul 10;14(7):426. doi: 10.3390/dj14070426 (PMC13408946; doi:10.3390/dj14070426)
Supplement: Supplementary file 1 [file dentistry-14-00426-s001.zip › dentistry-4285144-supplementary.pdf]

Table S1. Myotonometric measurements of the masseter muscle (right vs. left).

	**RMM**	**LMM**	**Difference RMM - LMM**			
	Mean ±SD	Mean ±SD	Mean	95% CI	p-value	SE
Control group						
Myoton F	14.68 ±3.01	14.92 ±2.42	-0.237	-0.870 ; 0.396	0.457	0.09
Myoton S	351.65 ±127.97	351.75 ±110.58	-0.100	-25.269 ; 25.069	0.994	0.00
Myoton D	1.68 ±0.26	1.71 ±0.25	-0.023	-0.078 ; 0.033	0.418	0.12
Myoton R	17.02 ±4.92	17.36 ±4.83	-0.337	-1.384 ; 0.710	0.522	0.07
Right PXB group						
Myoton F	14.61 ±2.27	14.66 ±2.44	-0.047	-0.603 ; 0.508	0.864	0.02
Myoton S	332.33 ±113.99	336.92 ±125.89	-4.583	-36.731 ; 27.564	0.774	0.04
Myoton D	1.66 ±0.28	1.69 ±0.28	-0.023	-0.104 ; 0.057	0.565	0.11
Myoton R	18.11 ±5.40	17.99 ±5.35	0.111	-1.109 ; 1.331	0.854	0.02
Left PXB group						
Myoton F	14.18 ±2.54	13.93 ±2.19	0.250	-0.132 ; 0.632	0.193	0.11
Myoton S	311.39 ±129.09	297.48 ±102.99	13.909	-11.146 ; 38.964	0.269	0.12
Myoton D	1.64 ±0.32	1.64 ±0.28	0.004	-0.062 ; 0.070	0.901	0.00
Myoton R	19.38 ±5.50	19.78 ±4.82	-0.405	-1.517 ; 0.708	0.467	0.08

Abbreviations: RMM: Right Masseter Muscle; LMM: Left Masseter Muscle; PXB: Posterior Crossbite; SD: Standard Deviation; CI: Confidence Interval; SE: Standard Error; F: Frequency (Hz); S: Stiffness (Nw/m); D: Decrement; R: Relaxation (ms);

Table S2. Myotonometric measurements of the anterior temporalis muscle (right vs. left).

	**RATM**	**LATM**	**Difference RATM - LATM**			
	Mean ±SD	Mean ±SD	Mean	95% CI	p-value	SE
Control group						
Myoton F	27.34 ±6.51	28.00 ±6.90	-0.655	-1.574 ; 0.264	0.159	0.10
Myoton S	740.72 ±131.24	748.90 ±151.10	-8.133	-36.749 ; 20.483	0.572	0.06
Myoton D	1.47 ±0.26	1.50 ±0.20	-0.034	-0.115 ; 0.048	0.411	0.13
Myoton R	8.00 ±1.65	8.10 ±1.50	-0.067	-0.390 ; 0.256	0.681	0.06
Right PXB group						
Myoton F	26.63 ±5.32	26.8 ±5.65	-0.150	-1.890 ; 1.590	0.862	0.03
Myoton S	747.69 ±96.72	749.19 ±115.03	-1.500	-34.600 ; 31.600	0.927	0.01
Myoton D	1.47 ±0.20	1.50 ±0.20	0.012	-0.052 ; 0.075	0.712	0.15
Myoton R	7.34 ±1.10	7.80 ±0.80	-0.431	-0.867 ; 0.006	0.053	0.48
Left PXB group						
Myoton F	24.01 ±4.90	27.7 ±25.9	-3.739	-11.689 ; 4.212	0.348	0.20
Myoton S	703.41 ±118.10	672.20 ±102.00	31.205	6.537 ; 55.872	0.014	0.28
Myoton D	1.49 ±0.18	1.40 ±0.20	0.055	-0.009 ; 0.118	0.091	0.47
Myoton R	8.32 ±3.42	8.50 ±2.90	0.182	-0.257 ; 0.620	0.408	0.06

Abbreviations: RATM: Right Anterior Temporalis Muscle; LATM: Left Anterior Temporalis Muscle; PXB: Posterior Crossbite; SD: Standard Deviation; CI: Confidence Interval; SE: Standard Error; F: Frequency (Hz); S: Stiffness (Nw/m); D: Decrement; R: Relaxation (ms)

Table S3. Comparison of electromyographic activity (RMS values) of the masseter muscle between right and left sides within each study group.

	RMM	LMM	Difference RMM - LMM			
	Mean ±SD	Mean ±SD	Mean	95% CI	p-value	SE
Control group						
Electro_ISO_RMS_mean	59.50 ±31.80	55.93 ±30.72	3.569	-2.865 ; 10.002	0.272	0.11
Electro_REP_RMS_mean	5.97 ±8.76	4.44 ±4.96	1.530	0.131 ; 2.929	0.033	0.22
Electro_ALG_RMS_mean	68.37 ±12.89	67.08 ±13.61	1.283	-2.344 ; 4.910	0.482	0.10
Electro_MAS_D_RMS_mean	38.16 ±21.63	23.28 ±16.82	14.878	10.392 ; 19.363	0.000	0.77
Electro_MAS_I_RMS_mean	28.29 ±18.96	33.37 ±22.64	-5.074	-9.170 ; -0.978	0.016	0.20
Right PXB group						
Electro_ISO_RMS_mean	57.98 ±32.60	54.39 ±29.90	3.593	-3.085 ; 10.272	0.282	0.12
Electro_REP_RMS_mean	6.24 ±5.33	8.38 ±16.11	-2.140	-6.821 ; 2.541	0.360	0.18
Electro_ALG_RMS_mean	68.61 ±11.35	68.24 ±11.83	0.368	-3.679 ; 4.415	0.855	0.03
Electro_MAS_D_RMS_mean	32.97 ±18.54	25.58 ±21.53	7.392	1.576 ; 13.209	0.014	0.37
Electro_MAS_I_RMS_mean	24.38 ±19.72	37.80 ±27.42	-13.427	-21.904 ; -4.950	0.003	0.56
Left PXB group						
Electro_ISO_RMS_mean	72.39 ±37.23	73.65 ±30.88	-1.261	-12.150 ; 9.628	0.816	0.04
Electro_REP_RMS_mean	8.24 ±7.57	7.16 ±5.77	1.077	-1.407 ; 3.560	0.387	0.16
Electro_ALG_RMS_mean	70.75 ±10.00	70.11 ±11.25	0.639	-2.484 ; 3.761	0.682	0.06
Electro_MAS_D_RMS_mean	44.12 ±22.90	37.40 ±24.39	6.720	-2.132 ; 15.572	0.133	0.28
Electro_MAS_I_RMS_mean	35.27 ±18.59	46.51 ±36.91	-11.247	-22.688 ; 0.194	0.054	0.39

Abbreviations: RMM: Right Masseter Muscle; LMM: Left Masseter Muscle; RATM: Right Anterior Temporalis Muscle; LATM: Left Anterior Temporalis Muscle; PXB: Posterior Crossbite; SD: Standard Deviation; CI: Confidence Interval; SE: Standard Error; ISO: maximum voluntary contraction; REP: muscular activity at rest; ALG: maximum voluntary contraction on cotton rolls; MAS_D: right side mastication; MAS_I: left side mastication

Table S4. Comparison of electromyographic activity (RMS values) of the anterior temporalis muscle between right and left sides within each study group.

	RATM	LATM	Difference RATM - LATM			
	Mean ±SD	Mean ±SD	Mean	95% CI	p-value	SE
Control group						
Electro_ISO_RMS_mean	71.67 ±39.62	72.42 ±40.50	-0.743	-10.415 ; 8.929	0.878	0.19
Electro_REP_RMS_mean	8.66 ±12.47	8.34 ±15.72	0.328	-3.997 ; 4.653	0.880	0.02
Electro_ALG_RMS_mean	66.14 ±12.49	69.98 ±12.05	-3.838	-7.478 ; -0.197	0.039	0.31
Electro_MAS_D_RMS_mean	36.30 ±30.44	28.07 ±22.63	8.229	0.481 ; 15.977	0.038	0.31
Electro_MAS_I_RMS_mean	35.36 ±46.70	34.26 ±20.32	1.104	-10.255 ; 12.462	0.847	0.03
Right PXB group						
Electro_ISO_RMS_mean	194.88 ±767.27	64.43 ±29.30	130.452	-131.605 ; 392.510	0.319	0.24
Electro_REP_RMS_mean	6.69 ±9.60	6.68 ±5.86	0.012	-3.074 ; 3.098	0.994	0.00
Electro_ALG_RMS_mean	64.70 ±15.72	68.28 ±14.17	-3.584	-8.661 ; 1.493	0.161	0.24
Electro_MAS_D_RMS_mean	39.35 ±89.61	20.07 ±12.76	19.283	-8.791 ; 47.356	0.172	0.30
Electro_MAS_I_RMS_mean	22.48 ±16.97	24.35 ±13.42	-1.869	-7.435 ; 3.698	0.500	0.12
Left PXB group						
Electro_ISO_RMS_mean	82.68 ±43.42	82.80 ±51.70	-0.113	-12.386 ; 12.160	0.985	0.00
Electro_REP_RMS_mean	7.68 ±6.34	10.36 ±11.82	-2.677	-6.087 ; 0.734	0.121	0.28
Electro_ALG_RMS_mean	72.27 ±12.08	68.46 ±10.63	3.810	0.556 ; 7.064	0.023	0.36
Electro_MAS_D_RMS_mean	31.36 ±23.31	32.83 ±27.65	-1.472	-6.954 ; 4.011	0.591	0.06
Electro_MAS_I_RMS_mean	29.80 ±18.82	36.40 ±29.17	-6.597	-14.103 ; 0.910	0.083	0.27

Abbreviations: RMM: Right Masseter Muscle; LMM: Left Masseter Muscle; RATM: Right Anterior Temporalis Muscle; LATM: Left Anterior Temporalis Muscle; PXB: Posterior Crossbite; SD: Standard Deviation; CI: Confidence Interval; SE: Standard Error; ISO: maximum voluntary contraction; REP: muscular activity at rest; ALG: maximum voluntary contraction on cotton rolls; MAS_D: right side mastication; MAS_I: left side mastication
